# Novel Maleic Acid, Crosslinked, Nanofibrous Chitosan/Poly (Vinylpyrrolidone) Membranes for Reverse Osmosis Desalination

**DOI:** 10.3390/ijms21197338

**Published:** 2020-10-04

**Authors:** Israr Ali, Muhammad Asim Raza, Rashid Mehmood, Atif Islam, Aneela Sabir, Nafisa Gull, Bilal Haider, Sang Hyun Park, Rafi Ullah Khan

**Affiliations:** 1 Department of Polymer Engineering & Technology, University of the Punjab, Lahore 54590, Pakistan; israrchem@gmail.com (I.A.); rajaan13@yahoo.com (R.M.); dratifislam@gmail.com (A.I.); aneela.pet.ceet@pu.edu.pk (A.S.); gullchemist@gmail.com (N.G.); rkhan.icet@pu.edu.pk (R.U.K.); 2 Advanced Radiation Technology Institute (ARTI), Korea Atomic Energy Research Institute, Jeongeup 56212, Korea; mohammadasimraza@yahoo.com; 3 Radiation Science and Technology, University of Science and Technology, Daejeon 34113, Korea; 4 Institute of Chemical Engineering & Technology, University of the Punjab, Lahore 54590, Pakistan; bilal.icet@pu.edu.pk

**Keywords:** electrospinning, reverse osmosis, desalination, permeation flux, salt rejection

## Abstract

Fresh and clean water is consistently depleting and becoming a serious problem with rapid increases in population, so seawater desalination technology has captured global attention. For an efficient desalination process, this work proposes a novel, nanofibrous, thin-film composite membrane (NF-TFC) based on the deposition of the nanofibrous active layer of a blend of chitosan (CS) and poly (vinylpyrrolidone) (PVP) crosslinked with maleic acid on a 3-triethoxysilylpropylamine functionalized cellulose acetate substrate. FTIR analysis demonstrated the development of chemical and physical interactions and confirmed the incorporation of functional groups present in the NF-TFC. Scanning electron microscopy (SEM) micrographs depict the fibrous structure of the active layers. The reverse osmosis (RO) desalination characteristics of NF-TFC membranes are elevated by increasing the concentration of the crosslinker in a CS/PVP blend. Cellulose acetate (CA)-S4 attained an optimal salt rejection of 98.3% and permeation flux of 42.9 L/m^2^h, suggesting that the NF-TFC membranes could be favorable for seawater desalination.

## 1. Introduction

Water is vital for the survival of all living organisms and human life, but over the past few decades, scarcity of fresh drinking water is becoming an alarming issue [1]. Approximately 97% of the total volume of water is available as sea water, while 2% is trapped by glaciers and icecaps. Only 0.8% is readily available as fresh and clean drinkable water [2]. Owing to the substantial rise in population, the clean water demand in 2030 will be increased to 6900 billion m^3^ from current consumption of water [3]. Sea water is abundantly available, which can fulfill this demand, but it is salty and inappropriate to drink. Many researchers have focused on desalination techniques to remove salts and other minerals from sea and brackish water [4]. Generally, the desalination process is comprised of two primary processes: (1) thermal processes, e.g., multistage flash, multiple effect distillation, vapor compression; and (2) membrane processes, e.g., electrodialysis (ED), membrane distillation (MD), and reverse osmosis (RO). In particular, RO is one of the prominent technologies for desalination, and is becoming pivotal and suitable because of its reliability, cost-effectiveness, energy efficiency, and salt rejection rate [5].

Recently, thin-film composite membranes (TFC) have gained enormous attention for use in RO and nanofiltration (NF), which provides the desired morphology for enhanced perm-selectivity, as well as high fouling resistance over a wide range of temperature and pH, with higher chemical and mechanical strength [6]. In polymeric membranes, cellulose acetate (CA) is primarily used as a substrate to provide spongy support and excellent mechanical strength, due to its excellent film forming characteristics, good transport properties, enhanced water affinity, and high hydrophilicity [7,8,9]. A promising silane base functionalizing agent 3-aminopropyltriethoxysilane (APTES) is added to enhance adhesion or adsorption of the active layer of CA substrate material to control the interfacial morphology. The typical linkage develops between amine (NH_2_) groups of APTES with hydroxyl (OH) groups of cellulose acetate (CA) units [10,11,12].

In general, various methods have been used to improve membrane separation performance by surface modification, blending, grafting, copolymerization, and interfacial polymerization. In recent years, the blending of polymers has become a simple and effective method for developing new materials that can tailor the characteristics of parent polymers and obtain a variety of properties into a new matrix. Polymer blending and crosslinking strongly influence permeability, selectivity, morphology, and degradation. In order to improve the physicochemical properties of a membrane, skinned active layer (nanofibrous) can be prepared from the blended miscible solution through electrospinning [13,14].

Chitosan (CS) is a naturally abundant polysaccharide, widely referred to as an excellent functional material, with many reactive hydroxyl and amino groups. The hydrophilic groups present on CS are considered to enhance the sorption of water, as well as the diffusion of small molecules in CS base membranes. CS is also used as a potential membrane material, due to its ease of modification, biocompatibility, non-toxicity, and film-forming ability [15]. In particular, the structural properties of polymer can be improved by crosslinking, annealing, and blending. Polyvinyl alcohol (PVA), polyethylene glycol (PEG), and poly (vinylpyrrolidone) (PVP) are synthetic polymers, are compatible, and can easily blend with other natural organic and inorganic compounds [16,17,18,19]. Islam and Yasin [20] blended CS with varying amount of PVA and crosslinked it with tetraethoxysilane (TEOS) to improve the thermal and mechanical stability of film, and it showed excellent resistance against acidic and basic media. Waheed et al. [21] prepared membranes of CA/PEG modified with CS by a two-stage phase inversion method. The performance of membranes showed that the salt rejection increased by 11% compared to the pristine CA/PEG membrane.

In the present work, the membranes were prepared in two steps, encompassing the formation of the substrate and the active layer. CA was used to prepare the substrate, and blends of CS and PVP crosslinked with maleic acid were prepared for the active layer via the electrospinning technique, in order to obtain a nanofibrous, thin-film composite membrane (NF-TFC). To the best of our knowledge, no previous work has been reported for the use of maleic acid as a crosslinker in RO membranes. The RO performance properties, permeation flux, salt rejection, chlorine resistance, short term stability test, membrane antifouling characteristics, surface morphology, and chemical interactions were investigated using techniques like dead-end RO permeation units, scanning electron microscopy (SEM), and Fourier transform infrared spectroscopy (FTIR).

## 2. Results and Discussion

### 2.1. FTIR Analysis

**Scheme 1:**
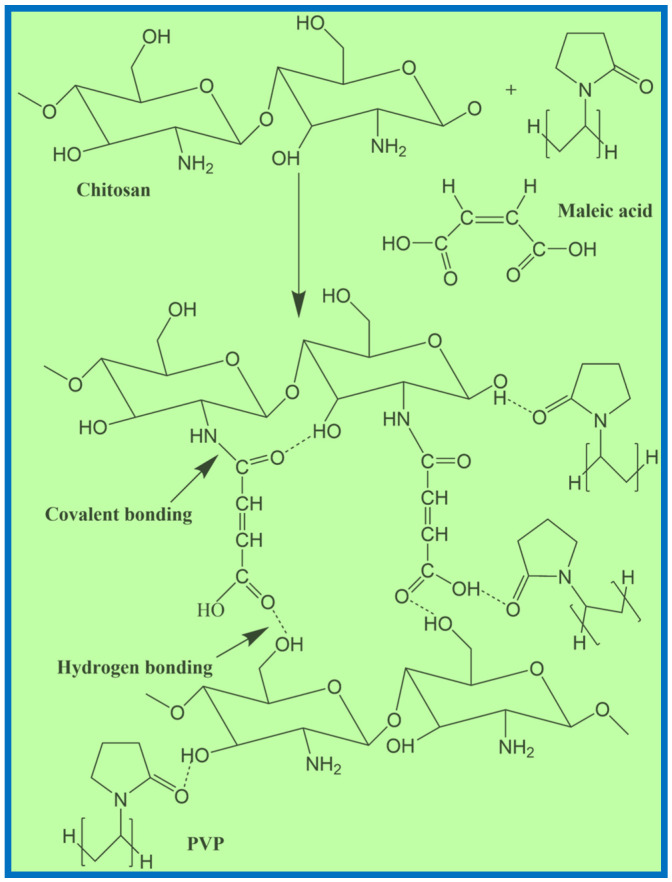
The proposed scheme showing the developed interactions in active layer of nanofibrous, thin-film composite membrane (NF-TFC).

**Figure 1:**
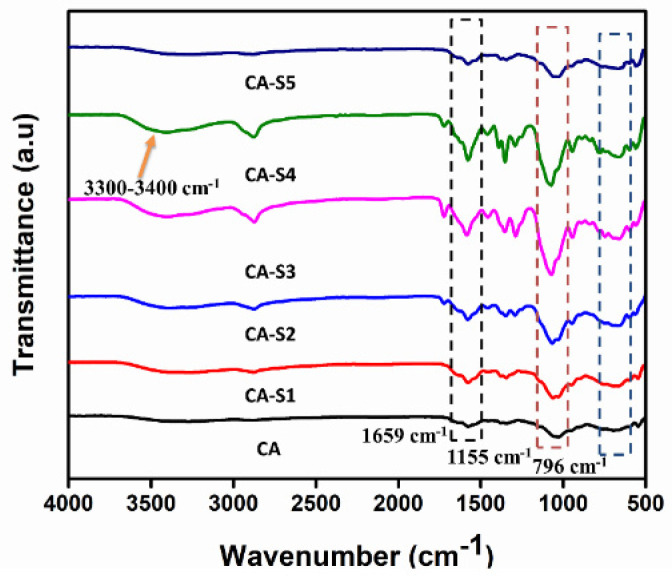
FTIR spectra of the cellulose acetate (CA), CA-S1, CA-S2, CA-S3, CA-S4, and CA-S5 membranes.

In terms of membrane characterization, FTIR analysis plays an essential role in detecting the intermolecular interactions between the polymers (CS, PVP) and crosslinkers. The infrared spectra of NF-TFC membranes were studied in the range of 4000–500 cm^−1^ as shown in Figure 1. The FTIR spectra shows bands at 893 and 1155 cm^−1^, and confirmed the pyranose ring and saccharine structure of chitosan [22,23]. The bands at 1653 cm^−1^ and 1322 cm^−1^ exhibited the characteristics of chitin and chitosan, respectively, and are reported as amide I and amide III peaks, respectively. A band at 1540 cm^−1^ corresponded to amide II of partially deacetylated chitosan. C–H and O–H bending, as well as C–N stretching overlapped in the range of 1400–1200 cm^−1^ with a sharp peak at 1390 cm^−1^, while the stretching bands of ether linkage of the pyranose ring of CS appeared unchanged [24]. A number of studies revealed that for pure PVP, the bands observed at 2924 cm^−1^ and 2892 cm^−1^, 1664 cm^−1^, 1461 cm^−1^, and 962 cm^−1^ were attributed to CH_2_ symmetric stretching, –C=O and C=C stretching, CH_2_ bending, and out-of-plane ring C–H bending, respectively and another band obtained at 1286 cm^−1^ showed characteristic vibrations of C–N in PVP. The spectra for CA and CA-S1 to CA-S5 membranes showed strong peaks at 1659 cm^−1^, which confirmed the presence of PVP in each blend [25,26]. A small new peak observed at 1714 cm^−1^ indicated the C=O vibrations of maleic acid, and the peaks shown at 827 cm^−1^ and 796 cm^−1^ are attributed to C–H rocking of the olefinic structure, confirming the incorporation of maleic acid on to the CS backbone [27]. A broad band at 3600–3200 cm^−1^ showed the existence of –NH symmetric vibration and ‒OH stretching vibration of inter- and intramolecular hydrogen bonding, as shown in Scheme 1.

### 2.2. Scanning Electron Microscopy

**Figure 2:**
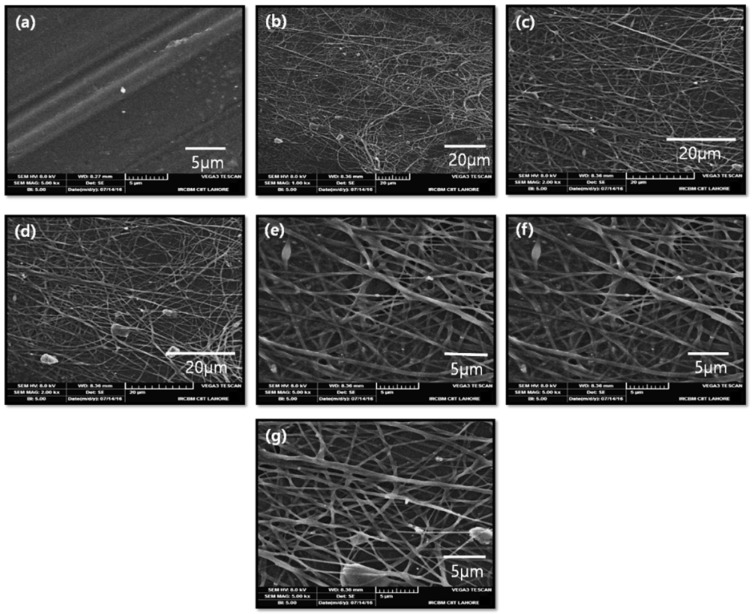
Scanning electron microscopy (SEM) micrographs of the (**a**) substrate, (**b**) control (CA), (**c**) CA-S1, (**d**) CA-S2, (**e**) CA-S3, (**f**) CA-S4, and (**g**) CA-S5 membranes.

The SEM images of the substrate, control (CA), and CA-S1 to CA-S5 are presented in Figure 2. The refined structure of electrospun nanofibers relies on the distance between tip of the needle and collector, flow rate of the blend solution, and the applied voltage. Additionally, the size of fibers is controlled by another important factor, which is significantly influenced by the concentration of the polymer solution. Figure 2 reveals that the uniform structure of the nanofiber matrix was formed without any bead formation on surface morphology. The increase in the concentration of crosslinkers in the polymer blend slightly changed the solution conductivity and significantly increased the viscosity of solution. The higher-viscosity solution greatly stabilized the chain entanglements of the polymer jet, which compensated for the influence of surface tension on the contraction of the diameter of jet. Thus, the higher viscosity of the spinning solution resulted in a decrease in the number of bead-shaped defects and fiber formation with higher average diameters.

### 2.3. Desalination Performance of Membranes

**Figure 3:**
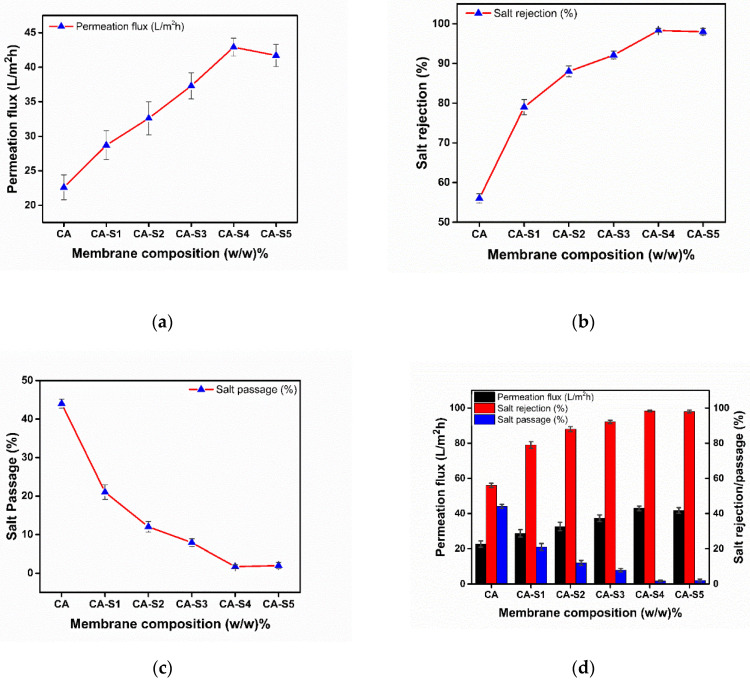
(**a**) Permeation flux (L/m^2^h) through an NF-TFC membrane. (**b**) Salt rejection (%) through an NF-TFC membrane. (**c**) Salt passage (%) through an NF-TFC membrane. (**d**) Combined graph of permeation flux (L/m^2^h), salt passage (%), and salt rejection (%) through an NF-TFC membrane.

With the addition of a crosslinker, the membrane develops a denser and tighter structure, because the polymer chains are intermingled and form a fibrous network [28]. These larger and complex structures hindered the free passage of salt molecules through the NF-TFC membranes. The performance of membranes prepared with varied concentration of maleic acid solution is shown in Figure 3, and the CA-S4 gave optimum results in terms of salt rejection and permeation flux, as compared to other samples. However, the resulting membrane revealed a suitable concentration for proper crosslinking. The solution diffusion model can be applied to understand the transport mechanism of NF-TFC membrane. In this model, the separation process occurs through the diffusive flow, when the permeation of the solvent and retention of the solute takes place in the membrane. According to this model, the transport process is comprised of three steps: starting from adsorption on the nanofibrous active layer of the membrane, then diffusion under pressure along the membrane, and lastly desorption [29,30]. In a NF-TFC membrane, the reactive amine and hydroxyl groups of CS make it hydrophilic. Additionally, PVP is also hydrophilic in nature, which plays an influential role in preferential water sorption and diffusion [31]. Maleic acid (crosslinker) consists of carboxyl groups (-COOH), which produce a charged surface on the membrane because they get readily ionized by the detachment of hydrogen and converted into negatively charged carboxylate ions (COO^−^). This uneven assortment of the charged species on the membrane surface results in the retention of the solute particles due to strong columbic forces. These electrostatic repulsive forces are responsible for salt rejection efficacy, commonly known as Donnan effect [32].

Primarily, the permeation flux and salt rejection were radically influenced by the concentration of a crosslinker. As the concentration of crosslinker increased, the salt rejection enhanced due to a higher degree of crosslinking. Incorporation of a crosslinker generated a structural compactness of the membrane because of strong interactions between the CS and PVP matrix, and formed a complex network structure. This nanofibrous packed structure hindered the free penetration of the salt molecules through the NF-TFC membrane, which resulted in the retention of salt. It was observed that with increase in amount of crosslinker from 0.5 to 2.5 wt %, salt rejection enhanced from 56 to 98.3%. As a result, the membrane showed excellent results because of its ability to hydrogen bond, as indicated in Scheme 1. The salt rejection and permeation flux are usually in reciprocation; hence, it appears to be an excellent finding that the inclusion of a crosslinker up to the optimal concentration enhanced both salt rejection and permeation flux. The results obtained from the present study showed the best performance of CA-S4, as it showed the highest salt rejection of 98.3%, a permeation flux of 42.9 L/m^2^h, and even after the chlorination test salt rejection remained at 90.0%. Also, Table 1 provides comparison of our work with other state-of-the-art published work.

### 2.4. Membrane Chlorine Resistance

**Figure 4:**
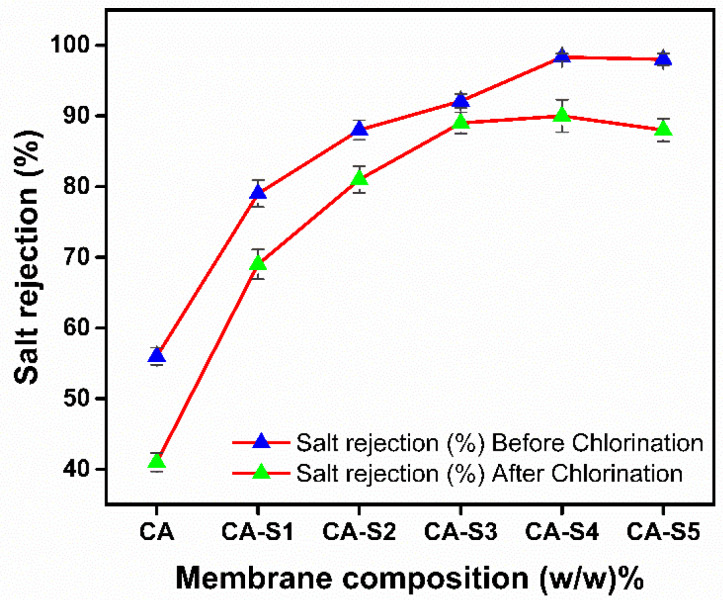
Salt rejection (%) through an NF-TFC membrane before and after chlorination.

The membrane resistance to chlorine exposure can be best explained by comparing the salt rejection of the RO membrane before and after chlorination. Figure 4 and Table 2 demonstrate that in the absence of crosslinker, the membrane showed low resistance to chlorine and salt rejection after chlorination decline from 56% to 41%; meanwhile, the difference between pre- and post-chlorination salt rejection was 15%. This denotes that the fibrous structure of the membrane was ruptured when it was exposed to the severe environment generated by the higher content of chlorine, while the incorporation of a crosslinker consequently improved chlorine resistance. As the concentration of crosslinker increased, the difference between the salt rejection pre- and post-chlorination became insignificant. The fabricated CA-S3 presented maximum impedance against chlorine because of the highest degree of crosslinking. The difference between salt rejection before and after chlorination was less than 3.1% of crosslinker-diminished chlorine resistance. The results indicate that the fibrous NF-TFC membrane successfully improved the chlorine resistivity. The enhanced stability was due to covalent bonding and hydrogen bonding between a crosslinker with CS and PVP. This minimized the decay of the polymer matrix by minimizing the number of target points where a chlorine atom would be adhered.

### 2.5. Stability Tests

**Figure 5:**
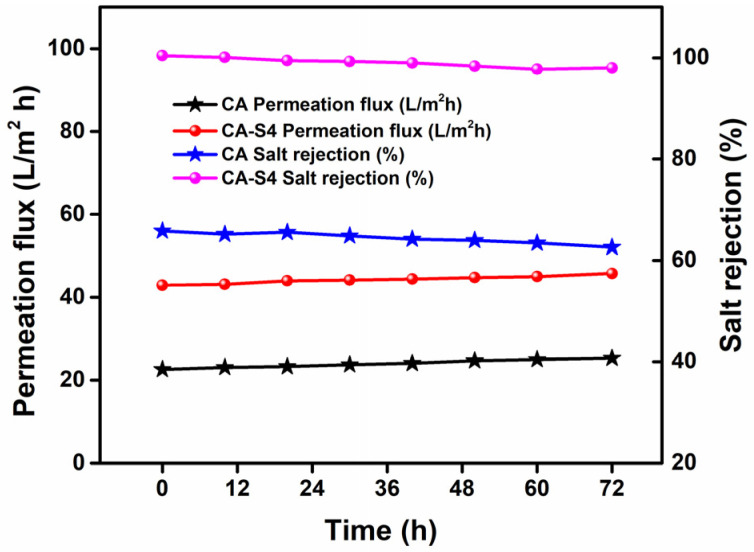
Short term stability of CA and CA-S4 NF-TFC membranes using NaCl 3.28 wt % concentration at a working pressure of 55.2 bar.

In RO membrane application, the stability test is considered to be the most significant analysis. A stability test of the NF-TFC membrane is shown in Figure 5. The stability of membrane was determined at a working pressure of 55.2 bar using NaCl solution, and permeation flux was noted after every 1 h for 72 h. The NaCl rejection did not considerably change, and the overall retention of NaCl salt decreased little and reached 95%. The experimental results showed that CA (control) and CA-S4 maintained the permeation flux after 72 h of operation, demonstrating the excellent stability of the membrane. These findings indicate that our membranes have a tendency to serve the function properly over the period of time.

### 2.6. Fouling Resistance

Fouling resistance is the fundamental characteristic to evaluate the performance of RO membranes. The fouling mechanism is related to the adsorption of model foulants on the surface of the membrane due to electrostatic interaction, van der Waals forces, and polarization between membrane materials with the foulants. In order to overcome the fouling problems, it is essential to decrease the interactions of foulants with the membrane surface. Long-term fouling resistance performance of NF-TFC membranes was evaluated on the dead-end filtration framework using model foulants humic acid and cetyl trimethylammonium bromide (CTAB). CA-S4 showed the best performance in terms of NaCl rejection and permeation flux, which is why CA-S4 and CA (control) were employed for the fouling test [40].

**Figure 6:**
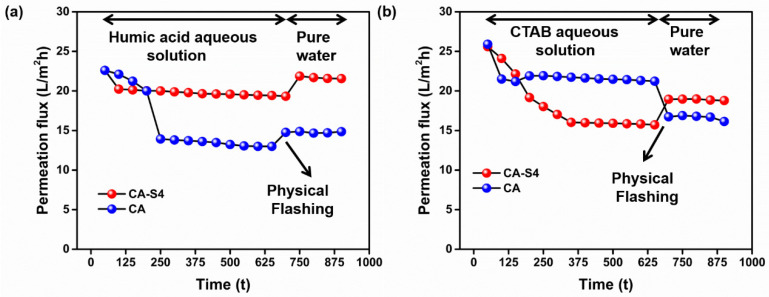
Time-dependent permeation flux (L/m^2^h) through an NF-TFC membrane using model foulants (**a**) humic acid and (**b**) cetyl trimethylammonium bromide (CTAB).

Figure 6a shows that model foulant humic acid enhanced the flux post flushing, as the negative charge is responsible for lowering the fouling effect. The rationale behind better fouling resistance of the membrane was due to less electrostatic interaction with the membrane surface, as well as steric hindrance of interconnected bulky groups of CS and PVP that were responsible for enhanced antifouling characteristics and cleaning capability. On the contrary, as shown in Figure 6b, the adhesion of fouling species decreased the flux profile after flushing. This results in the possible electrostatic interaction of cationic species of the CTAB foulants with the membrane surface, which has a slightly negative charge.

### 2.7. Contact Angle Determination

**Figure 7:**
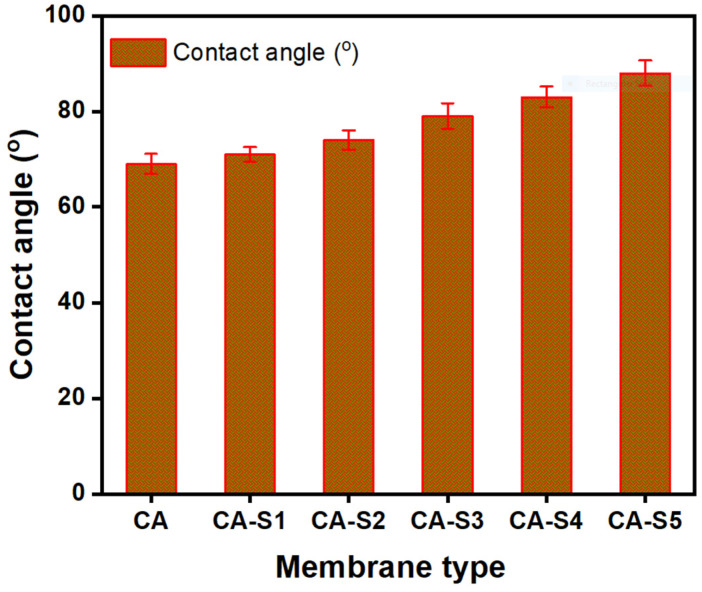
Contact angle of the membrane at ambient temperature.

The contact angle of membranes CA to CA-S5 are illustrated in Figure 7. It can be seen that the water contact angle of all prepared membranes was less than 90°, which indicates the hydrophilic nature of membranes. The contact angle is used to determine the non-covalent forces between the liquid used and the first monolayer of the surface [41]. The lower contact angle of 69° was shown by CA, while the contact angle increased in the crosslinked membranes from CA-S1 to CA-S5. The reported results showed that the membrane materials can be used as favorable materials to increase the hydrophilicity of membrane. The contact angle increased with increases in the amount of crosslinker from 0.5% to 2.5%. This is owing to a decrease in pore size with an increase in the percent amount of crosslinker, as less water can go inside the membrane, resulting in a higher contact angle; however, the angle is less than 90° in all membranes, which shows the hydrophilicity of membranes.

## 3. Materials and Methods

### 3.1. Materials

CA (39.0–40.3% acetyl content), PVP (K60, *M_w_* = 360,000 g/mol), CS (degree of deacetylation = 90%), sodium hypochlorite (6–14% effective chlorine basis solution), and APTES (99% purified) were obtained from Sigma Aldrich (Yongin, Korea). Reagents like dimethyl formamide (DMF), formic acid (analytical grade), absolute ethanol, TEOS (reagent grade, 98%), and analytical-grade maleic acid were procured from Sigma Aldrich (St. Louis, MO, USA). All solvents were used without further purification.

### 3.2. Preparation of NF-TFC Membrane

#### Preparation of CA (Cellulose Acetate) Substrate 

CA substrate was prepared by mixing 12.5 wt % CA in DMF solvent, keeping the temperature at 65 °C, and was continuously stirred for 2 h to form a homogeneous polymer solution. Functionalization and crosslinking of the substrate were obtained by adding TEOS and APTES (25 µL each in 3 mL of absolute ethanol) dropwise while stirring the blended solution. This process was continued for 2 h to accomplish proper mixing and crosslinking. Then, the solution was cast on the glass petri dish by a micrometer applicator (Sheen Instruments (117/300), Michigan, United States), achieving a thickness of ~0.45 mm, and was placed in lab scale oven (LVO-2040, Lab Tech, Korea) at 50 °C under controlled evaporation for 24 h. The CA substrate was then taken out by employing a sharp blade.

### 3.3. Preparation of Electrospinning Solutions

#### 3.3.1. CS/PVP Blends

CS (2.5 wt %) solution was prepared by dissolving 1 g CS in 40 mL of formic acid, stirring continuously, and filtering using a syringe filter (0.45 µm) to remove undissolved particles. This solution was diluted by adding 60 mL of ethanol, and 4 g PVP was added to prepare the co-dissolving spinning liquid. The weight ratio (1:4) of CS and PVP were blended together, and the total polymer concentration in the mixed solution was 20 wt %.

#### 3.3.2. Crosslinking of CS/PVP Blends with Maleic Acid

The prepared blends of CS/PVP were crosslinked by adding the maleic acid (crosslinker) with various quantities (0.5, 1.0, 1.5, 2.0, and 2.5% *w*/*v*), and stirred moderately at 70 °C until the solution obtained optimum viscosity. The control and solutions with various concentration of maleic acid are tabulated in Table 3, and listed as CA, CA-S1, CA-S2, CA-S3, CA-S4, and CA-S5, respectively. The above solutions were used to fabricate an active layer of nanofibers on APTES-functionalized CA substrate through the electrospinning unit.

#### 3.3.3. Electrospinning Process

An indigenously prepared electrospinning setup was used to fabricate the nanofibrous skinned layer of NF-TFC. In electrospinning process, the prepared blended solution was filled using a syringe with a metallic needle (stainless-steel), 1.5’ × 21 G, an OD of 0.82 mm, and an ID of 0.51 mm. The functionalized CA substrate was wrapped on the rotating cylindrical collector, and acted as cathode by applying the negative potential of a high-power voltage supply (~19 KV), while the needle of the syringe acted as an anode. Optimum electrospinning parameters were controlled as follows: the minimum distance between the collector and the tip of injection was maintained up to 12 cm, and the flow rate was adjusted at 0.5 mL/h. Then the blend accumulated on the substrate at 12.6 mL/m^2^. When polymeric fluid squeezed out from the slit of the needle, the high voltage charged the solution, thereby producing a repulsive force that reduced the surface tension of the fluid, and a jet erupted from the tip of the needle. The jet was stable near the tip, but the jet afterwards turned into bending instability [42]. As the charged polymer jet accelerated towards the collector, entanglements of polymer chain prevent the jet from breaking up, while the solvent evaporated and a smooth, defect-free nanofibrous layer was embedded onto the surface of substrate. An optimal nanofibrous film was put on the substrate surface, and finally it was separated, air-dried, and kept in self-sealing plastic bags.

### 3.4. FTIR Analysis

The FTIR spectra of membrane samples were scanned by using a Nicolet 6700 (Thermo Electron Corporation) assembled with an attenuated total reflectance (ATR) kit, and measured in the transmission mode at wave number range 4000–500 cm^−1^, and 128 scans per spectrum were recorded at a resolution of 4 cm^−1^.

### 3.5. Scanning Electron Microscopy

The surface morphology of fabricated membranes was determined through SEM (EO/JEOL model 6480, Tokyo, Japan) and scanned under an electron beam of high energy ranging from 0.2 to 40.0 KeV. In order to determine the diameter, fiber texture, and dimensions of the skinned layer of NF-TFC membrane, an accelerating high voltage of 15 kV was applied. For sample preparation, the test specimens were coated with platinum (Pt) vapor (Cressington Sputter Coater 108auto), and the images were obtained at different magnifications.

### 3.6. Reverse Osmosis Performance Evaluation

The RO performance and transport properties of the membranes were determined in terms of permeation flux and salt rejection (%), using a dead-end filtration framework, manufactured by stainless steel 316 (Model HP 4750 Stirred Cell, Sterlitech Corp., Kent, WA, USA). The fabricated membranes with effective area 14.6 cm^2^ (2.26 in^2^) were mounted into the RO cell. Commercially available solution of NaCl 3.28 wt % was fed into the nanofibrous layer of membranes, while the pressure was maintained 800 psi (55.2 bar). The permeation flux of NF-TFC membranes was evaluated in the permeation cell by calculating the volume of water streamed through the effective area of membrane in the unit time. It was calculated using Equation (1).

(1) $$F=\frac{V}{t\times A}$$

where *F* represents flux, *V* is the permeate volume, *t* represents time, and *A* is the effective area of membrane. A salinity meter (Traceable VWR, ISO 17025 Accredited) test was conducted to record the salt rejection in permeate and feed solutions [43,44].

### 3.7. Membrane Chlorine Resistance

It has been reported that membrane stability shows analogous effects when exposed to high chlorine content for a short time, as well as low chlorine content for a long time. Chlorine resistance tests were performed by exposing the NF-TFC membranes in a highly concentrated chlorine solution for a short time period. In order to prepare chlorinated solution (2000 mg/L) that was more oxidative and severe, the commercial NaClO solution (10 wt % chlorine contents) was diluted by distilled water and a small amount of 0.1 M HCl, to obtain a pH value of 4.0. Hence, to determine chlorine resistivity, NF-TFC membranes were subjected to 2000 mL sodium hypochlorite solution (NaClO) of pH 4.0 at room temperature for 2 h, and salt rejection was evaluated before and after the solution’s soaking into the membrane. All membranes were thoroughly washed with distilled water, the salt rejection (%) was determined thrice, and the average values are reported [45].

### 3.8. Antifouling Studies

Membrane fouling tests were carried out using a dead-end filtration framework with model foulants humic acid and cetyl trimethylammonium bromide (CTAB), which were used as representatives of anion and cation particles of solutes. The model foulants humic acid and CTAB were used in the feed solution (50 mg/L). The optimum performance membrane (CA-S4) was selected for this analysis, and the experiment was performed at an initial flux *J_wo_* of 25 L/m^2^ h and pH 7.0, and temperature was kept at 25 °C. In order to obtain the steady state flux *J_ws_*, time-dependent permeate flux *J_wt_* was recorded during the entire analysis. After completing the process, the CA-S4 membrane was washed with distilled water to eliminate the foulants that were deposited on the surface of membrane, and was reused for finding water flux *J_wc_*. The nature of the CA-S4 membrane was assessed on the basis of acquired data. The flux recovery ratio (FRR) and flux decline ratio (FDR) were calculated by Equations (2) and (3).

(2) $$\mathrm{FRR}\;\left(\%\right)=\frac{J_{wc}}{J_{wo}}\times 100$$

(3) $$\mathrm{FDR}\left(\%\right)=\left(J_{wo}-\frac{J_{wt}}{J_{wo}}\right)\times 100$$

### 3.9. Contact Angle Determination

The contact angle of membranes was measured using an SEO Phoenix 300 Touch (model: Phoenix 300T, Surface & Electro Optics Co., Ltd., Korea) using deionized water. Sample of 200 × 150 mm dimensions was kept on specimen stage of the instrument. Then, a 5 µL uniform drop of deionized water was poured on the sample. The measurements were taken until the drop completely disappeared. This procedure was repeated three times and the mean value was used.

### 3.10. Statistical Analysis

All the analyses were performed three times, and the obtained data was described as mean ± standard deviation (SD) by using Origin Pro 9.1 version software. Statistical significance was compared using a Student’s *t*-test. The values (*p* < 0.05) drawn were statistically significant.

## 4. Conclusions

In this study, a novel active layer of maleic acid, crosslinked CS/PVP membranes were synthesized by incorporating the various concentrations of maleic acid (crosslinker). In addition, the nanofibrous active layer has been fabricated on the APTES-functionalized CA substrate using an electrospinning setup, which was confirmed by SEM micrographs. Reverse osmosis performance properties, such as permeation flux, salt rejection, antifouling, short-term stability, and chlorine resistance were evaluated. The increased concentration of the crosslinker from 0.5% to 2.5% *w*/*v*, the salt rejection, and permeation flux of CA-S4 showed optimal values of 98.3% and 42.9 L/m^2^h, respectively. The reported results of CA-S3 rendered an excellent chlorine tolerance to the severe environment. NF-TFC membranes achieved valuable results for the reverse osmosis process, and they can pave the way for water desalination technology development. These entire findings recommend that the suggested NF-TFC membranes can be favorable for seawater desalination.

## Figures and Tables

**Table 1:** Comparison of salt rejection and permeation flux with the state-of-the-art published work.

Articles	Permeation Flux (L/m^2^h)	Salt Rejection (%)	Reference
PVA/NaAlg/ZnO–NPs	34.00	97.00	[33]
PVA/DGEBA/MWCNT	84.00	92.00	[34]
PU/PVC–co-VA	3.58 ± 0.38	96.00 ± 0.59	[35]
Modified PVA/Gum Arabic conjugate	94.00	98.00	[36]
GO	9.18	99.30	[37]
CA/TiO2(NPs)	0.42	76.10	[38]
CS/PVP	42.90	98.30	Present work
CA/PEG/FSP	1.312	78.00	[39]

**Table 2:** Salt rejection (%) through an NF-TFC membrane before and after chlorination.

Sample	Salt Rejection (%) Before Chlorine Stability Test	Salt Rejection (%) After Chlorine Stability Test
CA	56.0 ± 1.2	41.0 ± 1.3
CA-S1	79.0 ± 1.9	69.0 ± 2.1
CA-S2	88.0 ± 1.4	81.0 ± 1.9
CA-S3	92.1 ± 1.0	89.0 ± 1.5
CA-S4	98.3 ± 0.5	90.0 ± 2.3
CA-S5	98.0 ± 0.8	88.0 ± 1.6

**Table 3:** Weight ratios of chitosan (CS), poly (vinylpyrrolidone) (PVP), and maleic acid for varying membrane compositions.

Membranes	CS	PVP	Maleic Acid
	(g)	(g)	(g)
CA	1	4	-
CA-S1	1	4	0.05
CA-S2	1	4	0.1
CA-S3	1	4	0.15
CA-S4	1	4	0.2
CA-S5	1	4	0.25

## References

[1] Shannon M.A., Bohn P.W., Elimelech M., Georgiadis J.G., Marin B.J., Mayes A.M. (2008). Science and technology for water purification in the coming decades. Nature.

[2] Sachit D.E., Veenstra J.N. (2014). Analysis of reverse osmosis membrane performance during desalination of simulated brackish surface waters. J. Memb. Sci..

[3] Altaee A., Sharif A.O. (2011). Alternative design to dual stage NF seawater desalination using high rejection brackish water membranes. Desalination.

[4] Hochstrat R., Wintgens T., Kazner C., Melin T., Gebel J. (2010). Options for water scarcity and drought management—The role of desalination. Desalin. Water Treat..

[5] Charcosset C. (2009). A review of membrane processes and renewable energies for desalination. Desalination.

[6] Lau W.J., Ismail A.F., Misdan N., Kassim M.A. (2012). A recent progress in thin film composite membrane: A review. Desalination.

[7] Trache D., Thakur V.K. (2020). Nanocellulose and Nanocarbons Based Hybrid Materials: Synthesis, Characterization and Applications. Nanomaterials.

[8] Serbanescu O.S., Voicu S.I., Thakur V.K. (2020). Polysulfone functionalized membranes: Properties and challenges. Mater. Today Chem..

[9] Lee J.S., Heo S.A., Jo H.J., Min B.R. (2016). Preparation and characteristics of cross-linked cellulose acetate ultrafiltration membranes with high chemical resistance and mechanical strength. React. Funct. Polym..

[10] Stenina I., Golubenko D., Nikonenko V., Yaroslavtsev A. (2020). Selectivity of Transport Processes in Ion-Exchange Membranes: Relationship with the Structure and Methods for Its Improvement. Int. J. Mol. Sci..

[11] Bernd G.K.S., Lee D.W. (2020). Modular Chitosan-Based Adsorbents for Tunable Uptake of Sulfate from Water. Int. J. Mol. Sci..

[12] Taha A.A., Wu Y.N., Wang H., Li F. (2012). Preparation and application of functionalized cellulose acetate/silica composite nanofibrous membrane via electrospinning for Cr(VI) ion removal from aqueous solution. J. Environ. Manag..

[13] Zhang X.H., Liu Q.L., Xiong Y., Zhu A.M., Chen Y., Zhang Q.G. (2009). Pervaporation dehydration of ethyl acetate/ethanol/water azeotrope using chitosan/poly (vinyl pyrrolidone) blend membranes. J. Memb. Sci..

[14] Sudha K.M.K., Harish K.H.G., Chandramani R., Radhakrishna M.C. (2015). PVP Influence on PVA crystallinity and optical band gap. Arch. Phy. Res..

[15] Xu D., Hein S., Wang K. (2008). Chitosan membrane in separation applications. Mater. Sci. Technol..

[16] Shafiq M., Sabir A., Islam A., Khan S.M., Hussain S.N., Butt M.T.Z.Z., Jamil T. (2017). Development and performance characteristics of silane crosslinked poly(vinyl alcohol)/chitosan membranes for reverse osmosis. J. Ind. Eng. Chem..

[17] Ahmad A., Jamshed F., Riaz T., Sabad-E-Gul, Waheed S., Sabir A., Alanezi A.A., Adrees M., Jamil T. (2016). Self-sterilized composite membranes of cellulose acetate/polyethylene glycol for water desalination. Carbohydr. Polym..

[18] Sabir A., Shafiq M., Islam A., Jabeen F., Shafeeq A., Ahmad A., Zahid Butt M.T., Jacob K.I., Jamil T. (2016). Conjugation of silica nanoparticles with cellulose acetate/polyethylene glycol 300 membrane for reverse osmosis using MgSO_4_ solution. Carbohydr. Polym..

[19] Archana D., Singh B.K., Dutta J., Dutta P.K. (2015). Chitosan-PVP-nano silver oxide wound dressing: In vitro and in vivo evaluation. Int. J. Biol. Macromol..

[20] Islam A., Yasin T. (2012). Controlled delivery of drug from pH sensitive chitosan/poly (vinyl alcohol) blend. Carbohydr. Polym..

[21] Waheed S., Ahmad A., Maqsood S., Sabad-e-Gul, Jamil T., Islam A., Hussain T. (2014). Synthesis, characterization, permeation and antibacterial properties of cellulose acetate/polyethylene glycol membranes modified with chitosan. Desalination.

[22] Islam A., Imran Z., Yasin T., Gull N., Khan S.M., Shafiq M., Sabir A., Munawar M.A., Raza M.H., Jamil T. (2015). An investigation of ac impedance and dielectric spectroscopic properties of conducting chitosan-silane crosslinked-poly (vinyl alcohol) blended films. Mater. Res..

[23] Sokker H.H., Abdel Ghaffar A.M., Gad Y.H., Aly A.S. (2009). Synthesis and characterization of hydrogels based on grafted chitosan for the controlled drug release. Carbohydr. Polym..

[24] Islam A., Yasin T., Akhtar M.J., Imran Z., Sabir A., Sultan M., Khan S.M., Jamil T. (2016). Impedance spectroscopy of chitosan/poly(vinyl alcohol) films. J. Solid State Electrochem..

[25] Soundararajan A., Gomathi T., Sudha P.N. (2018). Preparation and characterization of chitosan-povidone blend with glutaraldehyde as crossliking agent. World J. Pharm. Res..

[26] Hasipoglu H.N., Yilmaz E., Yilmaz O., Caner H. (2005). Preparation and characterization of maleic acid grafted chitosan. Int. J. Polym. Anal. Charact..

[27] Marsano E., Vicini S., Skopińska J., Wisniewski M., Sionkowska A. (2004). Chitosan and poly(vinyl pyrrolidone): Compatibility and miscibility of blends. Macromol. Symp..

[28] Gohil J.M., Ray P. (2009). Polyvinyl alcohol as the barrier layer in thin film composite nanofiltration membranes: Preparation, characterization, and performance evaluation. J. Colloid Interface Sci..

[29] Lonsdale H.K., Merten U., Riley R.L., Jay J. (1965). Transport Properties of Cellulose Acetate Osmotic Membranes. J. Appl. Polym. Sci..

[30] Burghoff H., Lee K.L., Pusch W. (1980). Characterization of transport across cellulose-acetate membranes in the presence of strong solute-membrane interactions. J. Appl. Ploym. Sci..

[31] Zargar V., Asghari M., Dashti A. (2015). A Review on Chitin and Chitosan Polymers: Structure, Chemistry, Solubility, Derivatives, and Applications. ChemBioEng Rev..

[32] Malaisamy R., Mahendran R., Mohan D., Rajendran M., Mohan V. (2002). Cellulose acetate and sulfonated Polysulfone blend ultrafiltration membranes—I. Preparation and characterization. J. Appl. Polym. Sci..

[33] Raza M.A., Islam A., Sabir A., Ali I., Mehmood R., Bae J., Hassan G., Khan M.U. (2019). PVA/TEOS crosslinked membranes incorporating zinc oxide nanoparticles and sodium alginate to improve reverse osmosis performance for desalination. J. Appl. Polym. Sci..

[34] Falath W., Sabir A., Jacob K.I. (2016). Highly improved reverse osmosis performance of novel PVA/DGEBA cross-linked membranes by incorporation of Pluronic F-127 and MWCNTs for water desalination. Desalination.

[35] Ahmad A., Jamshaid F., Adrees M., Sagar S., Sabir A., Riaz T., Zaheer H., Islam A., Jamil T. (2017). Novel Polyurethane/Polyvinyl chloride-co-vinyl acetate crosslinked membrane for reverse osmosis (RO). Desalination.

[36] Falath W., Sabir A., Jacob K.I. (2017). Novel reverse osmosis membranes composed of modified PVA/Gum Arabic conjugates: Biofouling mitigation and chlorine resistance enhancement. Carbohydr. Polym..

[37] Chae H., Lee J., Lee C., Kim I., Park P. (2015). Graphene oxide-embedded thin-film composite reverse osmosis membrane with High flux, anti-biofouling, and chlorine resistance Graphene oxide-embedded thin-film composite reverse osmosis membrane with high flux, anti-biofouling and chlorine resistance. J. Memb. Sci..

[38] Shafiq M., Sabir A., Islam A., Khan S.M., Gull N., Hussain S.N., Butt M.T.Z. (2018). Cellulose acetate based thin film nanocomposite reverse osmosis membrane incorporated with TiO_2_ nanoparticles for improved performance. Carbohydr. Polym..

[39] Sabir A., Islam A., Shafiq M., Shafeeq A., Butt M.T.Z., Ahmad N.M., Sanaullah K., Jamil T. (2015). Novel polymer matrix composite membrane doped with fumed silica particles for reverse osmosis desalination. Desalination.

[40] Ayyavoo J., Nguyen T.P.N., Jun B.M., Kim I.C., Kwon Y.N. (2016). Protection of polymeric membranes with antifouling surfacing via surface modifications. Colloids Surf. A Physicochem. Eng. Asp..

[41] Sionkowska A., Wisniewska S.J., Planecka A., Kozlowska J. (2010). The influence of UV irradiation on the properties of chitosan films containing keratin. Polym. Degrad Stab..

[42] Reneker D.H., Yarin A.L. (2008). Electrospinning jets and polymer nanofibers. Polymer (Guildf.).

[43] Wang L., Li D., Cheng L., Zhang L., Chen H. (2011). Preparation of thin film composite nanofiltration membrane by interfacial polymerization with 3,5-diaminobenzoylpiperazine and trimesoyl chloride. Chin. J. Chem. Eng..

[44] Jahangiri F., Mousavi S.A., Farhadi F., Vatanpour V., Sabzi B., Chenari Z. (2016). Effect of CO_2_-laser irradiation on properties and performance of thin-film composite polyamide reverse osmosis membrane. Korean J. Chem. Eng..

[45] Sabir A., Falath W., Jacob K.I., Shafiq M., Munawar M.A., Islam A., Gull N., Butt M.T.Z., Sanaullah K., Jamil T. (2016). Hyperbranched polyethyleneimine induced polycationic membranes for improved fouling resistance and high RO performance. Eur. Polym. J..

